# Protocol update and statistical analysis plan for CADENCE-BZ: a randomized clinical trial to assess the efficacy of sodium benzoate as an adjunctive treatment in early psychosis

**DOI:** 10.1186/s13063-019-3232-8

**Published:** 2019-04-08

**Authors:** Carmen Lim, Andrea Baker, Sukanta Saha, Sharon Foley, Anne Gordon, David Ward, Bjorn Burgher, Frances Dark, Martin Beckmann, Stephen Stathis, George Bruxner, Alex Ryan, Drew Richardson, Sean Hatherill, Michael Berk, Olivia Dean, John McGrath, Cadence Working Group, Justin Chapman, Susan Patterson, Saveena Singh, Bryan Mowry, Linda Tracy, Michael Duhig, Amy Machin, Elizabeth Troman, Shuichi Suetani, Thomas Dinala, Lucinda Burton, Peter Cosgrove, Clare Gamble, James Scott

**Affiliations:** 1 Queensland Centre for Mental Health Research, The Park Centre for Mental Health, Wacol, Australia; 2 Queensland Brain Institute, University of Queensland, St Lucia, Australia; 3 Metro South Mental Health, 228 Logan Rd, Woolloongabba, QLD 4102 Australia; 4 Metro North Mental Health, Royal Brisbane and Women’s Hospital, Herston, Australia; 5 Metro North Mental Health Service, Prince Charles Hospital, Rode Rd, Chermside, 4032 QLD Australia; 6 Evolve Therapeutic Services Logan, Child and Youth Mental Health Services Logan, Academic Clinical Unit Logan, Metro South Hospital and Health Services, Logan, Australia; 7 Lady Cilento Children’s Hospital, Raymond Terrace, South Brisbane, QLD 4101 Australia; 8 Metro North Mental Health, Caboolture and Redcliffe Hospitals, Caboolture, QLD Australia; 9 UQ Centre for Clinical Research, Faculty of Medicine, The University of Queensland, Herston, QLD 4029 Australia; 10 Logan Hospital, Cnr Armstrong Rd and Loganlea Rd, Meadowbrook, QLD 4131 Australia; 11 IMPACT Strategic Research Centre, Deakin University, School of Medicine, Barwon Health, P.O. Box 291, Geelong, 3220 Australia; 12 Department of Psychiatry, University of Melbourne, Level 1 North, Main Block, Royal Melbourne Hospital, Parkville, 3052 Australia; 13 National Centre for Register-Based Research, Aarhus BSS, Aarhus University, Aarhus, Denmark

**Keywords:** Statistical analysis plan, Early psychosis, Randomized clinical trial, Sodium benzoate, PANSS, RCT

## Abstract

**Background:**

CADENCE-BZ is a multi-centre, parallel-group, double-blind randomized controlled trial designed to examine the clinical efficacy and safety of an accessible food preservative, sodium benzoate, as an add-on treatment for patients with early psychosis. The original study protocol was published in 2017. Here, we describe the updated protocol along with the Statistical Analysis Plan (SAP) for the CADENCE-BZ trial prior to study completion.

**Methods and materials:**

Two important changes were made to the original protocol: (1) improvements to our statistical analysis plan permitted a reduction in sample size; and (2) a revision in the secondary outcomes with the intent of reducing redundancy and excluding those measures that were not appropriate as outcomes.

**Conclusions:**

We provide the updated SAP prior to the completion of the study with the intent of increasing the transparency of the data analyses for CADENCE-BZ. The final participants are currently completing the study and the results will be published in the near future.

**Trial registration:**

Australian New Zealand Clinical Trials Registry (ACTRN12615000187549). Registered on 26th February 2015.

## Background

This paper provides the detailed statistical analysis plan (SAP) and outlines changes to the CADENCE–BZ study protocol. The original detailed protocol has been previously published [1]*.* CADENCE-BZ is a multi-centre, parallel-group, double-blind randomized controlled trial designed to examine the clinical efficacy and safety of an accessible food preservative, sodium benzoate, as an add-on treatment for patients with early psychosis. The trial was approved by the Metro South Hospital and Health Services Human Research Ethics Committee (HREC reference number HREC/14/QPAH/598). The primary objective was to determine if 12-week treatment with 1000 mg (500 mg twice daily (BD)) of sodium benzoate improves the Positive and Negative Syndrome Scale (PANSS) total score [2] compared to placebo.

The last participant was recruited in August 2018 with the final follow-up visit anticipated to be completed in October 2018. This protocol update and SAP were written to prevent outcome reporting bias [3], selective reporting [4], and data-driven results.

## Revised secondary outcomes

Eleven outcomes were listed in the original study protocol. This includes the primary outcome, the PANSS total score, and the secondary outcomes: (1) the Global Assessment of Functioning (GAF), (2) the Clinical Global Impression (CGI), (3) the Hamilton Depression Rating Scale (HDRS), (4) the Assessment of Quality of Life (AQoL), (5) the Opiate Treatment Index (OTI), (6) the Activity and Participation Questionnaire (APQ-6), (7) the International Physical Activity Questionnaire (IPAQ), (8) the Simple Physical Activity Questionnaire (SIMPAQ), (9) the Physical Activity Questionnaire (PAQ), and (10) the Patient Global Impression (PGI-I).

The following secondary outcomes will be excluded from the protocol: (i) APQ6, which is a measure of vocational activity and community participation—no studies have demonstrated the APQ6 has sensitivity to change [5]. By contrast, the AQoL, a 12-item measure of functioning and quality of life which was included in the original protocol, was used previously in a study of sodium benzoate in participants with chronic schizophrenia [6]. As both the AQoL and the APQ6 measure similar domains, it was decided to exclude the APQ6. (ii) IPAQ, SIMPAQ, PAQ—these three scales measure physical activity and were only collected at baseline. They are therefore unable to be used as outcomes and have thus been excluded. (iii) The Opiate Treatment Index (OTI) is a comprehensive evaluation tool that assesses drug and alcohol use over the previous month. Drug and alcohol use were considered as confounding variables rather than outcomes and the OTI was therefore excluded.

## Updated sample size and power

Our original sample size was based on two-sample *t*-test and utilised the conservative assumptions that there are few or no correlations between the baseline and follow-up measures, and that there are perfect correlations between follow-up measures [7]. Our sample size was reduced from 160 to 100 after we adjusted our SAP. We recalculated our sample size by revising our assumptions regarding the correlations for a variety of covariance structures and the number of repeated measures. This strategy has advantages from an ethical perspective (i.e., fewer patients need to be randomized, less exposure to potential adverse events or lack of efficacy) and with regard to cost-effectiveness (the study can be completed earlier). Using a two-tailed test with alpha set at 0.05 and 90% power, to detect a minimum clinical meaningful difference in total PANSS score of at least 5 units (SD = 14.3) between both treatment arms, we determined that we needed a sample of 39 persons in each group. This assumes the correlation between baseline and follow-up measurements is at least 0.6 and the correlation between various follow-up measurements is at most 0.5. Over a 12-week period, we predicted an attrition rate of 20%. Thus, we revised our sample size down to randomize 100 subjects and this amendment was recorded in the Australian New Zealand Clinical Trials Registry.

## Statistical analysis plan

### Data analysis and reporting

Data analysis and reporting will be done in accordance to the CONSORT guideline [8]. The CONSORT diagram comprising the number of participants who were screened, eligible, randomized, receiving their allocated treatment, withdrawn from/lost to follow-up along with reasons will be provided. All statistical tests will be carried out at the 5% (two-sided) significance level unless otherwise specified. Estimates along with 95% confidence intervals and associated *p* values will be reported. All analysis will be carried out using the SAS software version 9.4. Other software such as R may be used if required.

### Definition of intention-to-treat and per-protocol population

The analysis will be performed based on intention-to-treat. This will include all randomized participants who complied with the study protocol through the duration of the clinical trial, as well as participants who deviated from the study protocol. We defined treatment compliance as the average number of pills taken per day divided by daily protocol dosage (in this case, two pills per day). The analysis will be performed in the per-protocol population by including participants (1) that stayed on the trial for 12 weeks and (2) whose treatment compliance was ≥ 80% (3) with absence of major protocol violation.

### Missing values

A recent simulation study has revealed that mixed-effects model repeated measures (MMRM) analysis is robust in maintaining statistical properties of a test when compared to the multiple imputation method in handling missing values [9], assuming that participants dropping out of the study is unrelated to outcome conditioned on the study covariates (missing at random assumption). We will not be imputing missing data in the study outcomes.

### Comparison of characteristics at baseline

Comparisons of participants at baseline will be undertaken for the outcome measures and for the following characteristics: sex, age, living situation, waist circumference, height, blood pressure, and body mass index. Continuous data will be summarized by means and standard deviation. Categorical data will be summarized by number and percentages. Significant test (χ^2^, *t*-test) will be computed to compare both treatment groups for each baseline characteristic. These baseline characteristics will be included as a covariate in the multivariable analysis to account for any imbalance that may have occurred by chance between the treatment groups. It is recommended that these variables are adjusted in assessing the primary outcome [10, 11].

### Analysis of primary outcome

The primary outcome measure is the total score of PANSS. Primary outcome will be analysed using MMRM [12]. We will include treatment group, week, and treatment–week interaction as fixed effects and intercept as random effects in the model. We will use the first order autoregressive (AR) as the covariance structure of the MMRM model. The AR structure has the following characteristics: (1) it has homogenous variances and correlations that decline exponentially with time (i.e., two measurements that are in closer proximity in time are more strongly correlated (conditioned on *ρ*) than those that are more distant in time); and (2) it is appropriate when time periods are evenly spaced. We will also examine the residual to assess model assumptions and goodness-of-fit.

### Analysis of secondary outcomes

Table 1 provides a summary of methods of analysis for each secondary outcome. We will use the Bonferroni method [13] to appropriately adjust for the overall level of significance for multiple secondary outcomes. There are no pre-planned subgroup analyses.

**Table 1:** Variables/measures and method of analysis

Secondary outcomes Variable/outcome	Hypothesis	Outcome measure	Method of analysis
PANSS subscales	Improvement occurred	Score [continuous]	MMRM
GAF		Score [continuous]	MMRM
CGI		Scale [categorical]: Normal–moderate (1–4) vs severe (> 5)	Generalized linear mixed model (GLMM)
HDRS		Score [continuous]	MMRM
AQoL		Score [continuous]	MMRM
PGI-I		Scale [categorical]	Chi-squared test

### Safety outcomes

The number of treatment-related adverse events (AE) and serious adverse events (SAE) will be reported by their relationship as ‘definitely’, ‘probably’, and ‘possibly’ related to treatment. The number (and percentage) of participants with each AE/SAE will be presented for each treatment arm categorised by (1) preferred term (2), body system, (3) severity. The number (and percentage) of occurrences of each AE/SAE will also be presented for each treatment arm. No formal statistical testing will be undertaken.

## Conclusions

This update contains the SAP for the CADENCE-BZ trial to avoid the risk of outcome reporting bias and data-driven results. By publishing this where we pre-specify our methods and analyses, it is hoped that the results will be as robust and transparent as possible.

## Abbreviations

AQoL: Assessment of Quality of Life
AR (1): First order autoregressive
CGI: Clinical Global Impression (CGI)
CONSORT: Consolidated standards of reporting trials
GAF: Global Assessment of Functioning
GLMM: Generalized linear mixed model
HDRS: Hamilton Depression Rating Scale
IPAQ: International Physical Activity Questionnaire
MMRM: Mixed-effects model repeated measures
OTI: Opiate Treatment Index
PANSS: Positive and Negative Syndrome Scale
PAQ: Physical Activity Questionnaire
PGI-I: Patient global impression
SAP: Statistical analysis plan
SAS: Statistical analysis system
SIMPAQ: Simple Physical Activity Questionnaire
